# Quercetin Declines Apoptosis, Ameliorates Mitochondrial Function and Improves Retinal Ganglion Cell Survival and Function in *In Vivo* Model of Glaucoma in Rat and Retinal Ganglion Cell Culture *In Vitro*

**DOI:** 10.3389/fnmol.2017.00285

**Published:** 2017-09-07

**Authors:** Feng-Juan Gao, Sheng-Hai Zhang, Ping Xu, Bo-Qi Yang, Rong Zhang, Yun Cheng, Xu-Jiao Zhou, Wan-Jing Huang, Min Wang, Jun-Yi Chen, Xing-Huai Sun, Ji-Hong Wu

**Affiliations:** ^1^Eye Institute, Eye and ENT Hospital, College of Medicine, Fudan University Shanghai, China; ^2^State Key Laboratory of Medical Neurobiology, Institutes of Brain Science and Collaborative Innovation Center for Brain Science, Shanghai Medical College, Fudan University Shanghai, China; ^3^Shanghai Key Laboratory of Visual Impairment and Restoration, Science and Technology Commission of Shanghai Municipality, Shanghai, China; ^4^Key Laboratory of Myopia, Ministry of Health Shanghai, China

**Keywords:** quercetin, retinal ganglion cells, glaucoma, apoptosis, mitochondria, neuroprotection

## Abstract

Glaucoma is a progressive neuropathy characterized by the loss of retinal ganglion cells (RGCs). Strategies that delay or halt RGC loss have been recognized as potentially beneficial for rescuing vision in glaucoma patients. Quercetin (Qcn) is a natural and important dietary flavonoid compound, widely distributed in fruits and vegetables. Mounting evidence suggests that Qcn has numerous neuroprotective effects. However, whether Qcn exerts neuroprotective effects on RGC in glaucoma is poorly understood. In this study, we investigated the protective effect of Qcn against RGC damage in a rat chronic ocular hypertension (COHT) model *in* *vivo* and hypoxia-induced primary cultured RGC damage *in vitro*, and we further explored the underlying neuroprotective mechanisms. We found that Qcn not only improved RGC survival and function from a very early stage of COHT *in* *vivo*, it promoted the survival of hypoxia-treated primary cultured RGCs *in* *vitro* via ameliorating mitochondrial function and preventing mitochondria-mediated apoptosis. Our findings suggest that Qcn has direct protective effects on RGCs that are independent of lowering the intraocular pressure (IOP). Qcn may be a promising therapeutic agent for improving RGC survival and function in glaucomatous neurodegeneration.

## Introduction

Glaucoma is a progressive neuropathy characterized by the loss of retinal ganglion cells (RGCs), and it is a major cause of irreversible visual impairment worldwide, as damaged RGCs are incapable of repair or regeneration (Calkins, 2012). Strategies that delay or halt RGC loss have been recognized as potentially helpful for rescuing vision in glaucoma. Therefore, many studies have evaluated neuroprotection for glaucoma and many neuroprotective agents have been identified, such as N-methyl-D-aspartate (NMDA) receptor antagonists (memantine and brimonidine), glutamate release inhibitors (bis(7)-tacrine; Fang et al., 2010), calcium channel blockers (cilnidipine and lomerizine; Fitzgerald et al., 2009), neurotrophins (brain-derived neurotrophic factor and neurotrophic factor; Pease et al., 2009), antioxidants (astaxanthin and flavonoids; Yamagishi and Aihara, 2014), and others, which can be used alone or in combination with intraocular pressure (IOP)-lowering therapy. Despite intensive efforts and good laboratory evidence, these agents did not show significant efficacy in the clinic. Thus far, no neuroprotective drugs are available for glaucoma treatment (Van de Velde et al., 2015). It is imperative to identify more efficacious neuroprotective agents with potential clinical value for preventing or slowing down RGC loss as well as for preserving RGC function for multiple or, ideally, all mechanisms of glaucoma (Levin and Danesh-Meyer, 2010).

Quercetin (Qcn) is a natural and important dietary flavonoid compound that is widely distributed in fruits and vegetables. Mounting evidence suggests that Qcn has numerous beneficial effects, including anti-inflammatory (Periasamy et al., 2016), anti-apoptosis (Ben Salem et al., 2016), anti-ischemic (Ekinci Akdemir et al., 2016), anti-oxidation (Xu et al., 2016), anti-endoplasmic reticulum (ER) stress (Ben Salem et al., 2015), anti-mutagenic (Barcelos et al., 2011), and anti-viral (Wu W. et al., 2015) effects in addition to promoting mitochondrial biogenesis (Sharma et al., 2015). In the retina, it has been reported that Qcn has protective effects in multiple lesions, including retina ischemia-reperfusion injury (Arikan et al., 2015), oxidative damage of RPE cells (Hytti et al., 2015), diabetes-induced retinal neurodegeneration (Kumar et al., 2014), choroidal neovascularization in age-related macular degeneration (Zhuang et al., 2011), vascular endothelial growth factor-induced choroidal and retinal angiogenesis (Li et al., 2015), and ocular inflammation (Romero et al., 1989). However, whether Qcn exerts neuroprotective effects on RGC in glaucoma is poorly understood. The factors involved in RGC injury in glaucoma, such as oxidative stress, ischemia-reperfusion injury and glutamate excitotoxicity (Aihara, 2010), are all included in the mechanisms of cellular protection exerted by Qcn. Therefore, we speculated that Qcn may have a protective effect against RGC loss in glaucoma. To test our hypothesis, RGC function, viability, and apoptosis with or without Qcn treatment were investigated in a rat model of chronic glaucoma *in* *vivo* and hypoxia-induced primary cultured RGC damage *in vitro*.

## Materials and Methods

### Animals and Ethics Statement

We used Wistar rats (150–200 g) and newborn Sprague-Dawley rats (3 days; SLAC Laboratory Animal Co., Ltd. Shanghai, China) in this study. All animals received humane care. The study protocol was reviewed and approved by the animal experimental ethics committee of Fudan University. The animal handling and experimental protocols adhered to the approved guidelines of Animal Care and Use Committee of Fudan University and the Association Research in Vision and Ophthalmology (ARVO) Statement for the Use of Animals in Ophthalmic and Vision Research.

Wistar rats were randomly allocated into the following four groups: (1) the NC group (normal control group)-normal age matched, untreated rats; (2) Qcn group (Qcn treated group)—these normal rats received intravitreal injection of 2 μl of 10 μM Qcn (Sigma-Aldrich, St. Louis, MO, USA) 2 days before the day of commencement of the experiment and then once a week for 4 weeks. The contralateral eyes were treated with the same volume of saline as a sham control; (3) COHT group (chronic ocular hypertension group)—COHT was unilaterally induced in these rats by the injection of 5 μl of paramagnetic polystyrene microbeads (FluoSpheres; Invitrogen, Carlsbad, CA, USA; 15-μm diameter) to the anterior chamber of the right eye, as previously reported (Sappington et al., 2010). At the same time, the left eye was treated with the same volume of saline as sham control; and (4) COHT+Qcn group, (COHT rats treated with Qcn)—COHT was induced in these rats as described for group-3 (injection of 5 μl of paramagnetic polystyrene microbeads into the anterior chamber of the right eye) and treated with Qcn as for group 2 (intravitreal injection of 2 μl of 10 μM Qcn). The left eye was treated with the same volume of saline as sham control. In this study, all animals were anesthetized by intraperitoneal injection of chloral hydrate (300 mg/kg body weight).

### Intraocular Pressure (IOP) Elevation

IOP was measured in both eyes under general anesthesia once before paramagnetic polystyrene microbead injection, after 1 day, 3 days, at the end of 1st, 2nd 3rd and 4th week after microbead injection using a tonometer (Icare^®^ Tonolab, TioLat, Helsinki, Finland). IOP was always measured between 9–10 am by the same operator. Mean ± standard deviation (SD) of the middle four readings out of six valid rebound measurements was taken as IOP.

### Electroretinography (ERG) and Photopic Negative Response (PhNR) Recordings

The function of RGCs is impaired before death. It is crucial to identify early dysfunction of RGCs, before RGC loss. To assess the functional changes of RGCs in the early stage of COHT, we studied the function of whole retina and RGCs by electroretinography (ERG) and Photopic Negative Response (PhNR) at baseline and after 3, 7, and 14 days of IOP elevation, as previously reported (Rangaswamy et al., 2007; Porciatti, 2015). Each rat was dark-adapted for 1–2 h before recordings. After the rats were anesthetized, the pupils were dilated with phenylephrine hydrochloride and tropicamide. Light stimuli were delivered using a ColorDome unit on a green background with green light flashes. Recordings were generated using the Espion Visual Electrophysiology System (Espion E3, Diagnosys, Diagnosys UK Ltd, UK). Two such recordings were obtained for each eye at each time point and averaged. For the a-wave and b-wave of ERG and PhNR amplitudes, measurements were used as the difference between a peak and adjacent trough on the waveform.

### Retrograde Labeling of RGCs and Counting

Seven days before sacrifice, the rats were deeply anesthetized. Then, 2 μl of 5% of FluoroGold (FG; Sigma-Aldrich, St. Louis, MO, USA) was injected into the superior colliculus on each side, as previously reported (Wu et al., 2013). At euthanasia, the eyeballs were enucleated and directly fixed in 4% paraformaldehyde for 2 h at room temperature. The retinas were then carefully dissected and prepared as flatmounts. RGCs were quantified and averaged per eight microscopic fields of identical size using a laser scanning confocal microscope (TCS SP8, Hamburg, Germany) at a final magnification of 200×. The RGCs were manually counted by two operators who were blinded to the study using ImageJ software (NIH, Bethesda, MD, USA). The RGC density is expressed as the number of cells per mm^2^.

FG labeling indicates that the protective effect is most obvious at 2 weeks after Qcn administration, to further clarify the protective effect of Qcn on RGCs under COHT, we performed TUNEL and survivin staining on retinal cryosections at 2 weeks after COHT.

### Terminal Deoxynucleotidyl Transferase dUTP Nick End-Labeling (TUNEL) Assay

The TUNEL assay was performed according to the manufacturer’s protocol (*In Situ* Cell Detection Kit; Roche, Mannheim, Germany), as previously described (Wu J. H. et al., 2015). RGCs and retinal sections were fixed in 4% (w/v) paraformaldehyde at 4°C for 30 min. Subsequently, the TUNEL reaction mixture was added to the sample and maintained for 60 min at 37°C. The preparations were visualized using a confocal microscope (Leica SP8, Hamburg, Germany) and quantified using ImageJ software (NIH, Bethesda, MD, USA). Six microscope fields of view from six different wells were analyzed per treatment. The number of TUNEL-positive cells in ganglion cell layer (GCL) at a distance between 200 μm and 600 μm from the optic disc were counted. Only four sections were chosen from each eye, and each group contained three eyes.

### Immunofluorescence

Immunofluorescence staining was performed as is reported elsewhere (Wu et al., 2013). Rat eyes were sectioned at 10 μm; then, the sections were incubated in 0.1% Triton X-100 and 3% (w/v) bovine serum albumin (BSA) for 30 min, sequentially, at room temperature to prevent nonspecific background signal. The cryosections were then incubated with primary rabbit anti-survivin (1:200, Abcam, Cambridge, MA, USA) antibodies at 4°C overnight. The following day, the samples were incubated with fluorescein-conjugated goat anti rabbit secondary antibody (1:400, Molecular Probes, Waltham, MA, USA) and Hoechst staining. The stained sections were visualized and captured by confocal microscopy (Leica SP8, Hamburg, Germany).

### Cell Culture and Treatment

RGC isolation was performed as we described previously (Gao et al., 2016). Briefly, retinas were obtained from 1- to 4-day-old Sprague-Dawley rats and dissociated in 4.5 U/mL of papain solution (Worthington, Lakewood, NJ, USA). The cell suspensions were then sequentially incubated with a petri dish coated with rabbit anti-macrophage antibody (Cedarlane Laboratories, Ontario, ON, Canada) and mouse anti-Thy1.1 antibody (Abcam, Cambridge, MA, USA). RGCs were collected and seeded into appropriate plates coated with mouse-laminin (Trevigen Inc., Gaithersburg, MD, USA) and poly-D lysine (Sigma-Aldrich, St. Louis, MO, USA). The RGC purity was approximately 85% (Gao et al., 2016). RGCs were then incubated with 200 μM cobalt chloride (CoCl_2_, Sigma-Aldrich, St. Louis, MO, USA) to induce hypoxia and apoptosis 48 h after seeding (Kim et al., 2013); then, 0, 1, 10, 20, 50 or 100 μM Qcn was added for 24 h, or the optimal concentration was given for 48 h.

### Cell Counting Kit-8 Assay for RGC Viability

RGCs were seeded into 96-well plates and treated with CoCl_2_ or/and Qcn for 24 h or 48 h. Then, 10 μl of CCK8 solution (Dojindo Laboratories, Kumamoto, Japan) was added to each well, and the samples were incubated at 37°C for 4 h before analysis at 450 nm with a Tecan Genios (Synergy H1, BIOtAK). All values are expressed as the mean ± SD of at least three wells and at least three separate experiments.

### LDH Release

After each treatment, all supernatant media was collected to evaluate the lactate dehydrogenase (LDH) release from the cytoplasm of damaged RGCs. The assay was performed using an LDH cytotoxicity detection kit (Promega, Fitchburg, WI, USA) according to the manufacturer’s instructions. Briefly, 50 μl of reconstituted substrate mix (Promega LDH kit) was added to each sample; after incubation at 25°C in the dark for 30 min, the enzymatic reaction was stopped with 50 μL of stop solution (Promega LDH kit). Absorbance was measured at 490 nm using a microplate reader (Synergy H1, BIOtAK). All experiments were performed in triplicate.

### Flow Cytometric Analysis for Apoptosis

The proportion of apoptotic cells was measured using fluorescence-activated cell sorting (FACS) on a FACSCalibur according to the instructions in the Annexin V-FITC/propidium iodide (PI) flow cytometric assay kit (Becton Dickinson, San Jose, CA, USA). Briefly, after each treatment, cells were trypsinized and stained with Annexin V-FITC and PI at room temperature for 20 min in the dark according to the manufacturer’s protocol. Then, stained cells were analyzed using FACS to differentiate the percentage of cells in early apoptosis (Annexin V+/PI−) and late apoptosis (Annexin V+/PI+). All experiments were performed in triplicate.

### Western Blot Analysis

Cell protein extraction and Western blot analysis were performed as previously reported (Wu J. H. et al., 2015; Gao et al., 2017). Briefly, cultured RGCs were lysed, and total proteins were extracted on ice with cell lysis buffer (Cell Signaling Technology, Boston, MA, USA) and protease inhibitor cocktail (Sigma-Aldrich, St. Louis, MO, USA). The protein concentration was quantified using a BCA protein assay kit (Thermo Fisher Scientific, Rockford, IL, USA). Equal amounts of protein were separated by SDS-polyacrylamide gel electrophoresis and then transferred to 0.22-μm PVDF membranes. After blocking with 5% non-fat milk for 1 h, the membranes were incubated overnight at 4°C with primary antibodies against B cell lymphoma 2 (Bcl-2, Abcam, Cambridge, MA, USA), cleaved caspase-3 antibody (Abcam, Cambridge, MA, USA) and β-actin (Abcam, Cambridge, MA, USA). Signals were monitored by the Kodak Imaging System (Kodak 440CF) using ECL Western blot substrate (Hyperfilm ECL, Thermo Fisher Scientific, Rockford, IL, USA). Then, they were quantified by densitometry using ImageJ software (NIH, Bethesda, MD, USA).

### Measurement of the Mitochondrial Membrane Potential (∆ψm)

The mitochondrial membrane potential (∆ψm) was evaluated using MitoProbe JC-1 dye (Invitrogen, Carlsbad, CA, USA) as previously described (Zhang et al., 2016). In brief, RGCs with different treatments were incubated with JC-1 at 37°C for 30 min while protected from light and assessed via FACS (Becton Dickinson, San Jose, CA, USA) and a confocal fluorescence microscope (Leica; green: 488 nm excitation/530 nm emission; red: 550 nm excitation/590 nm emission). The JC-1 monomer (green) and J-aggregate (red) were separately detected in FL1 (green fluorescence, *x*-axis) and FL2 (red fluorescence, *y*-axis) channels. Quantitative analysis was performed using ImageJ software, and ∆ψm was indicated by the ratio of the mean red fluorescence to the mean green fluorescence.

### Measurement of Reactive Oxygen Species (ROS)

To detect the reactive oxygen species (ROS) level, RGCs were incubated with 10 μM dihydroethidium (DHE) for 30 min in the dark. Fluorescence was observed with a confocal fluorescence microscope and quantified by FACS using the PE channel.

### Statistical Analyses

All data are expressed as the mean ± SD. The distributions of the amplitudes and inter-event intervals between the events were compared using the Kolmogorov–Smirnov test. Statistical analysis was performed using one-way analysis of variance (ANOVA) with Bonferroni’s multiple comparision test (using Prism 5.0 (Graph Pad Software Inc, San Diego, CA, USA). A *P* value < 0.05 was considered the threshold for significance.

## Results

### IOP Elevation

**Figure 1:**
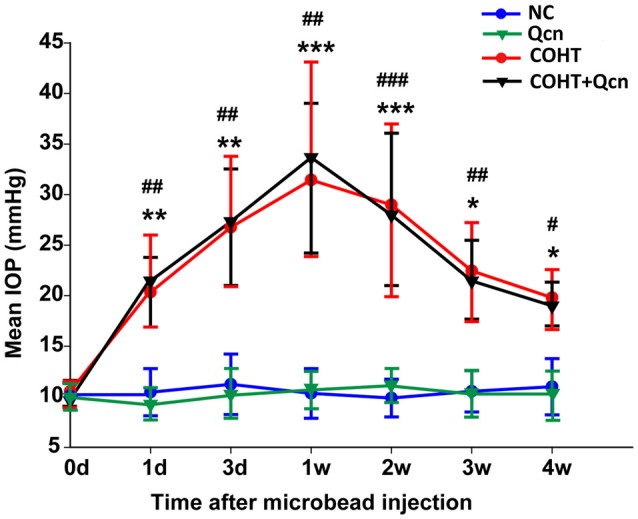
Mean intraocular pressure (IOP) of rats in the NC, quercetin (Qcn), chronic ocular hypertension (COHT) and COHT+Qcn groups. IOP was observed from 0 weeks to 4 weeks after microbead injection. The data are given as the mean ± standard deviation (SD); ^#^*p* < 0.05, ^##^*p* < 0.01 and ^###^*p* < 0.001 for the COHT group compared with the NC group. *p < 0.05, **p < 0.01 and ***p < 0.001 for the COHT+Qcn group compared with the Qcn and NC groups.

IOP was measured in each rat prior to injection (basal) and every 3 days after injection with a rebound tonometer (Figure 1). There was no significant difference between the mean basal IOPs (10.57 ± 0.59 mm Hg) of the four groups (*p* > 0.05). As expected, microbead injection induced a sustained elevation in IOP, as previously reported (Samsel et al., 2011). The average IOP of the microbead-injection eyes ranged from 18.99 ± 2.34 to 33.67 ± 9.05 mmHg at 1 day to 4 weeks after injection, which was significantly higher than that of the corresponding control eyes (*p* < 0.05). The IOP at each point after Qcn administration did not differ from the corresponding non-Qcn-treated eyes (*p* > 0.05). These results demonstrated that COHT is effectively induced by the anterior chamber injection of microbeads in rats, which is similar to human ocular hypertension and chronic glaucoma. In addition, Qcn administration had no effect on IOP.

### Qcn Ameliorates RGC Dysfunction in the COHT Rat Model

**Figure 2:**
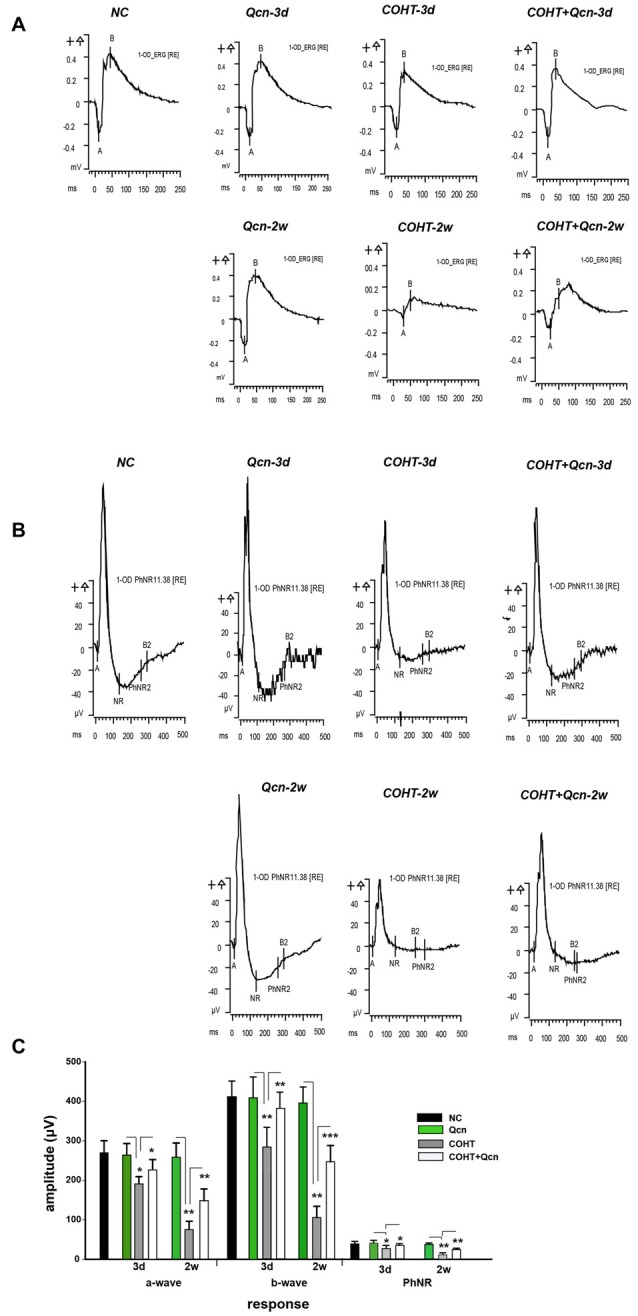
Effects of Qcn on electroretinography (ERG) and Photopic Negative Response (PhNR) responses. **(A)** Representative ERG at baseline, 3 days and 2 weeks of COHT with or without Qcn. **(B)** Data analyses of ERG a-wave, b-wave and PhNR amplitudes for the NC, Qcn, COHT and COHT+Qcn groups after 3 days and 2 weeks of microbead injection. **(C)** PhNR amplitudes at baseline, 3 days and 2 weeks of COHT with or without Qcn. The data are expressed as the mean ± SD, *p < 0.05, **p < 0.01 and ***p < 0.001 for the COHT group compared with the NC group and COHT+Qcn group compared with the COHT group.

The a-wave and b-wave amplitudes of the COHT group were only slightly smaller and slower than that of the NC group at an early stage of COHT (3 days, *p* < 0.05, Figures 2A,B), while the PhNR amplitude was significantly reduced by approximately 55% (*n* = 16, *p* < 0.01, Figure 2C), indicating that RGC function was impaired prior to that of other retinal cells, such as photoreceptor cells. However, the amplitudes of a-waves, b-waves and PhNR were significantly depressed by 72.17% (*p* < 0.01), 69.43% (*p* < 0.01) and 71.92%, respectively, at the 2nd week after COHT (*p* < 0.01), suggesting that retinal function, especially RGC function, is markedly impaired with the extension of COHT. Qcn administration significantly attenuated the reduction of PhNR amplitudes from a very early stage after COHT, by 54.10 ± 3.25% at the third day (*p* < 0.01) and 72.45 ± 4.56% at the 2nd week (*p* < 0.01). However, the amplitudes of a-waves and b-waves showed a delayed recovery, which was reversed by 0.97 ± 0.12- and 1.34 ± 2.35-fold at the 2nd week after COHT (*p* < 0.01). Qcn injection did not affect the electroretinographic response, as no significant difference in a-waves, b-waves and PhNR amplitude were observed between Qcn group and NC group (*p* > 0.05, Figures 2A,B).

### Qcn Promotes RGC Survival

**Figure 3:**
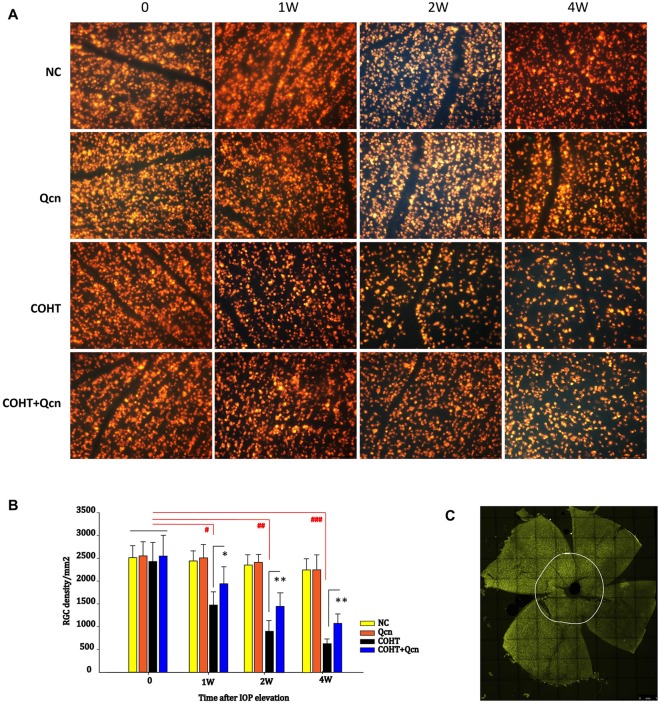
FluoroGold (FG) labeling of surviving retinal ganglion cells (RGCs) in flat-mounted retinas in the NC, Qcn, COHT and COHT+Qcn groups after 0, 1, 2 and 4 weeks of microbead injection. All images were captured at same magnification. Scale bar, 100 μm **(A)**. **(B)** Quantification of FG-labeled RGCs. The data are given as the mean ± SD; ^#^*p* < 0.05, ^##^*p* < 0.01 and ^###^*p* < 0.001 and *p < 0.05, **p < 0.01. **(C)** Whole flat-mounted retina. The point on the white circle shows the central position selected for the RGC count.

To assess whether Qcn could increase RGC survival under COHT, RGCs were detected by retrograde FG labeling (Figures 3A,C). In the eyes before microbead injection and in the NC group, the mean RGC density was 2435 ± 397 cells/mm^2^. After COHT, RGC somas were lost over time (Figures 3A,B), with a reduction of 39.4% (*p* < 0.05), 67.12% (*p* < 0.01) and 73.84% (*p* < 0.001) at the 1st, 2nd and 4th weeks, respectively. After 1, 2 and 4 weeks of Qcn treatment, the mean RGC density recovered, increasing by 19.18 ± 3.24%, 24.14 ± 5.22% and 15.89 ± 4.37%, respectively, which were significantly increases compared with the densities in the corresponding COHT group (*p* < 0.05). There was no significant difference in the RGC density between the NC and Qcn groups (*p* > 0.05). These results suggested that the intravitreal delivery of Qcn could promote RGC survival under COHT.

**Figure 4:**
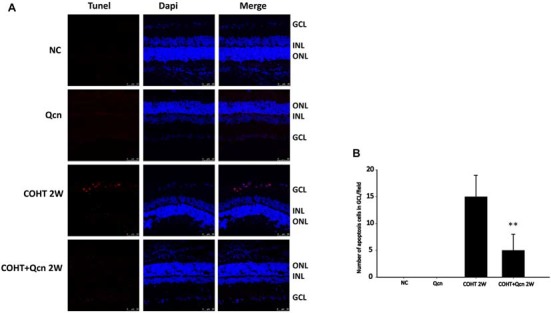
**(A)** Terminal Deoxynucleotidyl Transferase dUTP Nick End-Labeling (TUNEL) analysis of retinas from four different treatment groups (NC, Qcn, COHT and COHT+Qcn groups) at 2 weeks after microbead injection. Red indicates TUNEL-positive cells, and blue indicates DAPI. GCL, retinal ganglion cell layer; INL, inner nuclear layer; and ONL, outer nuclear layer. Scale bar, 50 μm. **(B)** Quantitative analysis of TUNEL-positive cells in retinas from different treatment groups. The data are presented as the mean ± SD. **p < 0.01.

**Figure 5:**
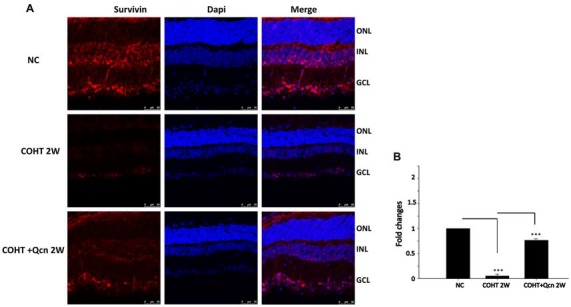
**(A)** Immunofluorescence of survivin staining (red) of retinas from three different groups at 2 weeks after microbead injection. The NC group (top), COHT (middle) and COHT+Qcn group (bottom). Red indicates positive survivin staining; blue indicates DAPI; GCL, retinal ganglion cell layer; INL, inner nuclear layer; and ONL, outer nuclear layer. Scale bar, 50 μm. **(B)** Quantitative analysis of survivin-positive regions in retinas from different treatment groups. The data are presented as the mean ± SD, ***p < 0.001.

To further clarify the protective effect of Qcn on RGCs under COHT, we performed TUNEL and survivin staining on retinal cryosections at 2 weeks after COHT. Consistent with previous reports (Can et al., 2015), we found a significant increase in TUNEL-positive cells in the retinal GCL of retinas with COHT compared with those in the NC group (Figures 4A,B). However, the number of TUNEL-positive cells was significantly reduced by two-fold when Qcn was administered (*p* < 0.01). There was no significant difference in the TUNEL-positive cells between the NC and Qcn groups (*p* > 0.05). Survivin, an inhibitor of apoptosis (Zhou et al., 2016), was identified by immunofluorescence analysis (Figure 5A). Survivin expression in the GCL and inner nuclear layer (INL) was significantly down-regulated by 94.81% after 2 weeks of COHT compared with the expression in the NC group (Figures 5A,B, *p* < 0.001), while Qcn treatment remarkably reversed its expression by 11.86 ± 2.52-fold compared with that in the corresponding COHT group (*p* < 0.001).

### Qcn Protects Against Hypoxia-Induced RGC Apoptosis

**Figure 6:**
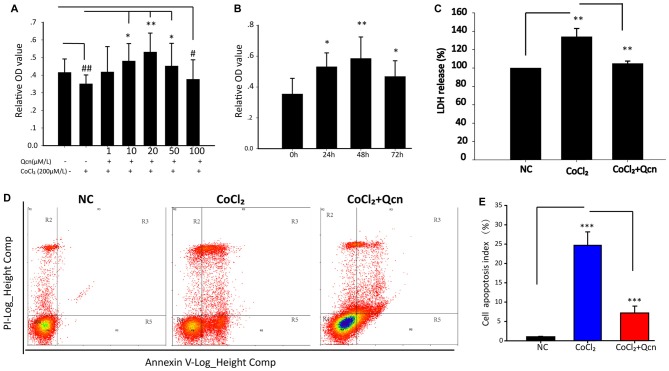
**(A)** The cell viability of RGCs treated with 0–100 μM Qcn with or without CoCl_2_ was measured by the CCK-8 assay. Bars indicate the mean ± SD; ^#^*p* < 0.05 and ^##^*p* < 0.01 compared with the NC group. *p < 0.05 and **p < 0.01 compared with the CoCl_2_ without Qcn group. **(B)** RGC viability when treated with 20 μM Qcn and 200 μM CoCl_2_ for 0, 24, 48 and 72 h. Bars indicate the mean ± SD. *p < 0.05 and **p < 0.01 compared with 0 h. **(C)** The impact of Qcn on lactate dehydrogenase (LDH) release of RGC treated with 20 μM Qcn and 200 μM CoCl_2_ for 48 h. Bars indicate the mean ± SD. **p < 0.01 when the hypoxia group was compared with the NC and CoCl_2_+Qcn groups. **(D)** Cell apoptosis detected by AnnexinV and propidium iodide (PI) staining in RGCs treated with 20 μM Qcn and 200 μM CoCl_2_ for 48 h. The R4 quadrant (Annexin V−/PI−), R5 quadrant (Annexin V+/PI−) and R3 quadrant (Annexin V+/PI+) indicate the percentages of viable cells, apoptotic cells and necrotic cells, respectively. **(E)** The percentage of apoptotic cells after CoCl_2_ treatment with or without Qcn. The values represent the mean ± SD of three independent experiments. ***p < 0.001.

To further explore the underlying neuroprotective mechanism, primary RGCs were cultured under hypoxia with or without Qcn. CoCl_2_ was used to induce hypoxia and mimic the glaucomatous micro-environment *in vitro*, as previously reported (Du et al., 2013). To achieve this goal, 1 μM, 10 μM, 20 μM, 50 μM or 100 μM Qcn was added to RGCs with 200 μM CoCl_2_ (Wang et al., 2015), the RGC viability was analyzed using the CCK8 assay. As shown in Figure 6A, hypoxia significantly inhibited cell viability (*p* < 0.01), and Qcn treatment markedly increased RGC survival when the concentrations of Qcn were 10 μM (*p* < 0.05), 20 μM (*p* < 0.01) and 50 μM (*p* < 0.05) compared with the survival seen in the hypoxia group. However, the number of RGCs was significantly decreased at 100 μM Qcn (*p* < 0.05), indicating that Qcn had toxicity at high concentrations. There can be several reasons for this. First, when the drug concentration is too high, the osmotic pressure of the medium increases, which may do damage to RGCs. Second, it is reported that Qcn concentrations higher than 50 μM can led to decreased mitochondrial function of retinal pigment epithelial cells (Kook et al., 2008). Furthermore, too high Qcn doses may activate other molecular mechanisms or generate de novo proteins, this requires further studies. Therefore, 20 μM was used as the optimal effective concentration for subsequent studies. Then, RGCs were incubated with 20 μM Qcn under hypoxia conditions for 24 h, 48 h and 72 h. Our results showed that the RGC viability exhibited a time-dependent increase with Qcn incubation (*p* < 0.05) and reached optimum levels at 48 h (*p* < 0.01) compared with that in the hypoxia group (Figure 6B).

**Figure 7:**
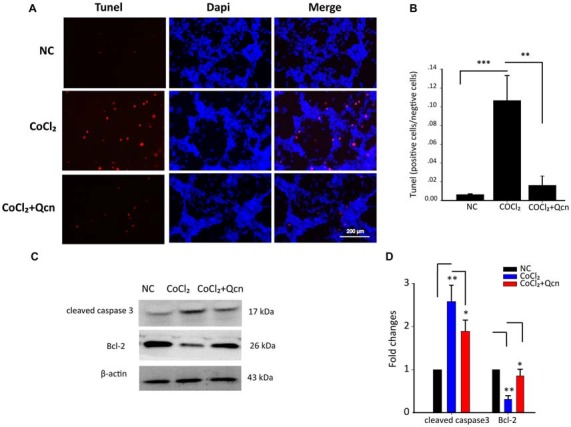
**(A)** TUNEL analysis of RGCs from the following three different groups: NC group, CoCl_2_-treatment group (48 h) and CoCl_2_ with Qcn-treatment group (48 h). Red indicates TUNEL-positive cells, and blue indicates DAPI. All images were captured at the same magnification. Scale bar, 200 μm. **(B)** Quantitative analysis of TUNEL-positive cells in retinas from different treatment groups. The data are presented as the mean ± SD; **p < 0.01 and ***p < 0.001. **(C,D)** Expression of cleaved caspase-3 and Bcl-2 detected by Western blot in RGCs from the following three treatment groups: NC group, CoCl_2_-treatment group (48 h) and CoCl_2_ with Qcn-treatment group (48 h). The bars indicate the mean ± SD. β-actin was used as an internal control. *p < 0.05 and **p < 0.01.

Furthermore, the protective effect of Qcn was evaluated by lactate dehydrogenase (LDH) released into culture media (Figure 6C). In the presence of hypoxia, LDH release from RGCs increased by 31 ± 4.36% compared with that in the NC group (*p* < 0.01); however, the effect was reversed with 20 μM Qcn (*p* < 0.01). Then, RGC apoptosis and death were evaluated by flow cytometry-based Annexin V+PI assay and TUNEL staining. As shown in Figures 6D,E, 7A,B, the numbers of Annexin V (+) and TUNEL-positive RGCs were significantly increased under hypoxia (23.33 ± 4.78-fold vs. 17.98 ± 4.34-fold, respectively; *p* < 0.01), whereas Qcn-treatment remarkably decreased the number of apoptotic RGCs (decreases of 90.02 ± 5.06% vs. 83.48 ± 4.33%, *p* < 0.01). These results collectively indicate that Qcn protected RGCs from hypoxia-induced apoptosis *in* *vitro*, which is similar to what we observed *in* *vivo*.

### Mechanisms of the Qcn Protective Effect on RGCs

For further in-depth exploration of the mechanisms of the protective effect of Qcn on RGCs under hypoxia, the apoptotic protein levels of Bcl-2 and cleaved caspase-3, the ∆Ψm and ROS generation were analyzed. Western blot analysis demonstrated that the expression levels of the anti-apoptotic protein Bcl-2 were reduced, while the pro-apoptotic protein cleaved caspase-3 levels, the activated form of caspase 3, were significantly upregulated after 48 h of CoCl_2_ treatment (Figures 7C,D, *p* < 0.01). However, these changes were remarkably reversed by Qcn treatment (Figures 7C,D, *p* < 0.05). These data indicated that Qcn could protect against hypoxia-induced RGC apoptosis by upregulating the expression of Bcl-2 and suppressing the level of cleaved caspase-3.

**Figure 8:**
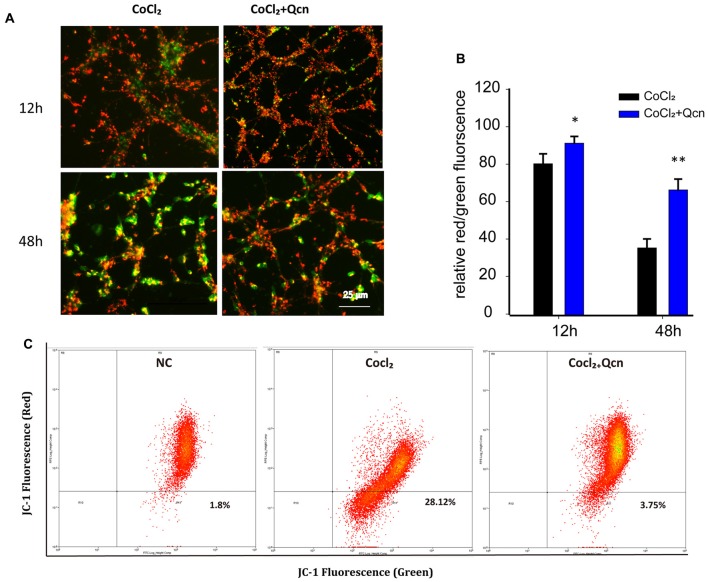
**(A)** Representative images of JC-1 staining of RGCs with CoCl_2_ and Qcn treatment for 12 h and 48 h. All images were captured at the same magnification. Scale bars, 100 μm. **(B)** Quantitative analysis of the ∆ψm by the ratio of the red and green fluorescence. The bars indicate the mean ± SD. *p < 0.05 and **p < 0.01 for the hypoxia+Qcn group compared with the hypoxia group. **(C)** Fluorescence density of J-aggregates (*y*-axis) against JC-1 monomers (*x*-axis) displayed in a dot plot.

**Figure 9:**
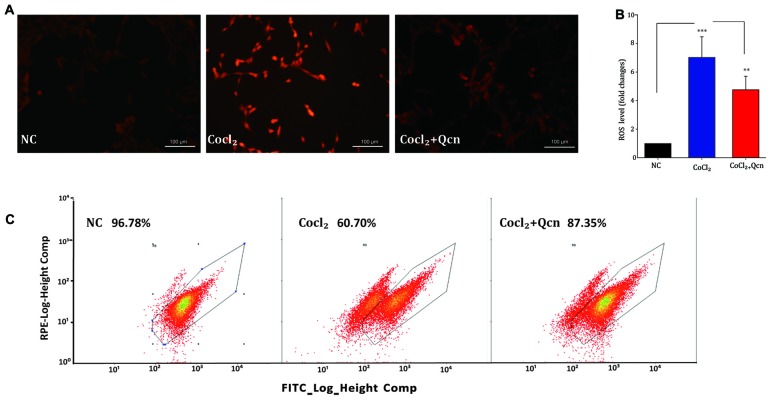
**(A)** Reactive oxygen species (ROS)-induced fluorescence was visualized by confocal microscopy. Scale bar, 100 μm. **(B)** Quantitative analysis of ROS fluorescence intensities in RGCs from different treatment groups. The data are presented as the mean ± SD, **p < 0.01 and ***p < 0.001. **(C)** Flow cytometry analysis of ROS generation shows a leftward-shift in the log of FITC in the CoCl_2_-treatment group, whereas Qcn treatment effectively alleviates the shift.

Qcn has been shown to play a fundamental role in modulating mitochondrial function by influencing ∆Ψm (Ben Salem et al., 2016). To determine whether Qcn also has an anti-apoptotic effect on hypoxia-induced RGC by ameliorating mitochondrial function, ∆Ψm and intracellular ROS levels were determined under hypoxia with or without Qcn. Quantitative data demonstrated that Qcn treatment preserved the ∆Ψm by 13.58 ± 2.65% (at 12 h, *p* < 0.05) and 86.51 ± 6.86% (at 48 h, *p* < 0.01) compared with that in the control group (Figures 8A,B). Measurement of the fluorescence of the aggregate and monomer forms of JC-1 by flow cytometry further supported this conclusion (Figure 8C). Increased ROS levels are closely related to mitochondrial dysfunction. As shown in Figures 9A,B, a significant increase in red fluorescence representing ROS production was observed after CoCl_2_ treatment; the increase was approximately 7.33 ± 1.58-fold (*p* < 0.001). Qcn treatment dramatically reduced the ROS generation compared with that in the CoCl_2_-treatment group (*p* < 0.01). Flow cytometry analysis shows a leftward-shift in the log of FITC and red fluorescence in the CoCl_2_-treatment group, while Qcn treatment effectively reduced CoCl_2_-induced ROS production (Figure 9C). These findings suggest that the protective effects of Qcn are mediated, at least in part, by the direct prevention of CoCl_2_-induced loss in ∆Ψm and through an antioxidant mechanism of scavenging ROS.

## Discussion

Numerous studies have confirmed that Qcn has multiple biological activities and enormous potential for clinical application with safety (Sun et al., 2016). Qcn is currently undergoing clinical trials for treating cancer and is very likely to become a promising drug of choice in the near future (Madaan et al., 2016). However, as far as we know, no report is available for the use of Qcn on glaucomatous neuroprotection. Therefore, we investigated the protective effect of Qcn against RGC damage using a rat COHT model *in* *vivo* and explored the underlying molecular mechanism by hypoxia-induced primary cultured RGC damage *in vitro*. We found that Qcn could preserve RGC function as well as prevent RGC apoptosis in a rat model of chronic glaucoma *in* *vivo* and hypoxia-induced RGC apoptosis *in vitro*. The mechanism does not depend on a decrease in IOP; instead, it occurs via ameliorating mitochondrial function and preventing mitochondria-mediated apoptosis.

Qcn was administered by oral or intraperitoneal injection in previous studies on the retina (Kumar et al., 2014; Arikan et al., 2015). However, to maintain an effective drug concentration and prolong the exposure of Qcn to retina after a single administration, intravitreal injection was chosen as the mode of Qcn administration in the current study. We found that this local administration was both well-tolerated and effective. Additionally, a single administration reduces animal suffering, which is supported and advocated by the Declaration of Helsinki on the care and use of animals (Villar, 1988).

There is compelling evidence that RGC dysfunction occurs early, is progressive, and precedes RGC soma loss in glaucoma (Shou et al., 2003). Therefore, early functional protection is crucial for slowing the progression of glaucomatous optic neuropathy. Although numerous neuroprotective agents have been observed to inhibit at least some RGC soma loss (surviving RGCs may not be functional) in glaucoma models (Guo et al., 2006), few studies have linked the functional improvement of these agents to RGC. PhNR is a slow negative component of the photopic full-field ERG that follows the b-wave, and it can capture RGC function throughout the entire visual field, providing a direct, objective assessment of the functional changes of RGCs (Preiser et al., 2013; Porciatti, 2015; Wilsey and Fortune, 2016). In the current study, we found that Qcn could preserve the PhNR wave early from 3 days of COHT, indicating that early RGC functional damage could be delayed or even rescued by Qcn administration with COHT. Apoptosis occurs following functional damage. FG retrograde labeling showed that Qcn effectively reduced the loss of RGCs without IOP reduction, which was further verified by TUNEL and survivin staining. Collectively, these results demonstrate that Qcn plays a protective role on RGC function and survival with COHT *in vivo*.

Apoptosis is tightly controlled by a variety of signaling pathways. Bcl-2, an anti-apoptotic protein localized to the mitochondria, is a central regulator of the intrinsic apoptotic pathway, which is also called the mitochondrial pathway (Hardwick and Soane, 2013). Bcl-2 primarily regulates cell death via its effects on mitochondrial outer membrane permeabilization (Pradelli et al., 2010), which controls the release of cytochrome C from the mitochondria to cytoplasm. The accumulation of cytochrome C in the extracellular space consequently activates caspase 3 and related downstream proteins, which eventually leads to apoptosis. Moreover, Qcn mediates the protective effects via regulating the expression of cleaved caspase-3 and Bcl-2, which has been reported by numerous studies (Kumar et al., 2014). In this context, we questioned whether these pathways were also involved in the anti-apoptotic effects of Qcn in hypoxia-induced RGCs. Our results showed that Bcl-2 expression was dramatically increased, while cleaved caspase-3 was remarkably decreased in Qcn-treated RGCs compared with the levels in the hypoxia-induced group. Bcl-2 overexpression can inhibit the accumulation of cytochrome C in the cytoplasm to inhibit cell apoptosis (Wu and Bratton, 2013). Therefore, one possible neuroprotective mechanism of Qcn is that it inhibits RGC apoptosis partially via increasing Bcl-2, while subsequently down-regulating cleaved caspase-3 expression, which is consistent with previous studies (Hu et al., 2015). ∆Ψm is a valuable indicator of mitochondrial functional status in living cells, and the lipophilic cation JC-1, a sensitive and non-invasive specific probe, is currently the gold standard for rapidly measuring ∆Ψm (Brooks et al., 2013). In this study, we found that Qcn treatment effectively recovered the ∆Ψm of RGC under hypoxia, indicating that Qcn has a protective role on mitochondrial function, which has been verified in several other types of cells and tissues, such as pancreatic β-cells (Carrasco-Pozo et al., 2016), the frontal cortex, hippocampus (Nichols et al., 2015; Gupta et al., 2017), mouse livers (Yu et al., 2016) and others. Mitochondria are the major sites of ROS production under physiologic conditions, and ROS generation is associated with mitochondrial dysfunction (Tezel, 2006). According to our data, Qcn attenuates CoCl_2_-induced ROS formation, which further supports that Qcn modulates mitochondrial function to protect RGCs. Taken together our results demonstrated that Qcn exerts neuroprotective effects on hypoxia-induced RGC apoptosis via ameliorating mitochondrial function and preventing mitochondria-mediated apoptosis.

A variety of molecular signals—acting alone or in cooperation—have been involved in glaucomatous pathophysiology, including oxidative stress (Chen et al., 2015), glutamate excitotoxicity (Lam et al., 1999), mitochondrial dysfunction (Nickells, 1999), glia activation (Lam et al., 2009), inflammation (Levkovitch-Verbin, 2015), autophagy (Deng et al., 2013), ischemia (Almasieh et al., 2012), ER stress (Ha et al., 2015), and others, while Qcn has been reported to have many benefits and medicinal properties with regard to these pathological processes. Therefore, we propose that Qcn may offer protection to RGC with COHT via a variety of mechanisms. Further studies are needed to elucidate the exact mechanism by which Qcn protects RGCs with COHT.

In conclusion, this study provides the first direct evidence that Qcn preserved RGC function and prevented RGC apoptosis in a rat model of chronic glaucoma *in* *vivo* and in hypoxia-induced RGC apoptosis *in vitro*. The mechanism does not depend on a decrease in IOP but instead involves ameliorating mitochondrial function and preventing mitochondria-mediated apoptosis. Our results provide important evidence that Qcn may be a promising therapeutic strategy for ameliorating RGC damage in glaucomatous neurodegeneration.

## Author Contributions

J-HW and X-HS designed this work, revised it critically and finally approved the version to be published. PX, RZ, B-QY, X-JZ, J-YC, YC, W-JH and MW took part in some of the experimental studies, for example, western blotting, PCR analysis and cell culture. F-JG and S-HZ drafted, revised the manuscript and took part in a majority of the work. All authors read and approved the manuscript.

## Conflict of Interest Statement

The authors declare that the research was conducted in the absence of any commercial or financial relationships that could be construed as a potential conflict of interest.
